# Neutrophil to lymphocyte ratio in Alzheimer’s disease: A systematic review and meta-analysis

**DOI:** 10.1371/journal.pone.0305322

**Published:** 2024-06-25

**Authors:** Aynaz Mohammadi, Mohammad Mohammadi, Mostafa Almasi‐Dooghaee, Omid Mirmosayyeb

**Affiliations:** 1 School of Medicine, Iran University of Medical Sciences, Tehran, Iran; 2 Neurology Department, Firoozgar Hospital, School of Medicine, Iran University of Medical Sciences, Tehran, Iran; 3 Firoozgar Clinical Research Development Center (FCRDC), Iran University of Medical Sciences, Tehran, Iran; 4 Isfahan Neurosciences Research Center, Isfahan University of Medical Sciences, Isfahan, Iran; 5 Department of Neurology, School of Medicine, Isfahan University of Medical Sciences, Isfahan, Iran; University of Missouri, UNITED STATES

## Abstract

**Background:**

The Neutrophil-to-Lymphocyte Ratio (NLR) is a clinical indicator of peripheral inflammation that is easily accessible. It is worth noting that the formation of amyloid-β (Aβ) plaques and neurofibrillary tangles has been linked to inflammation and immune dysregulation. The main objective of this systematic review and meta-analysis is to comprehensively evaluate the existing body of research concerning the NLR in the context of Alzheimer’s disease (AD) and mild cognitive impairment (MCI).

**Method:**

We conducted a comprehensive online search and included studies that evaluated the NLR in 1) patients with AD or MCI and 2) healthy control (HC) participants. We also pooled mean and standard deviation (SD) data for each group.

**Results:**

Ultimately, 12 studies encompassed 1,309 individuals diagnosed with AD with mean NLR levels of 2.68, 1,929 individuals with MCI with mean NLR levels of 2.42, and 2,064 HC with mean NLR levels of 2.06 were included in this systematic review and meta-analysis. The mean NLR was 0.59 higher in AD patients compared to HC participants (mean difference (MD) = 0.59 [0.38; 0.80]). Similarly, the mean NLR was higher in AD than MCI patients (MD = 0.23 [0.13; 0.33]). Additionally, the mean NLR was higher in individuals with MCI compared to HC participants (MD = 0.37 [0.22; 0.52]). In the subgroup meta-analysis based on the Mini-Mental State Examination (MMSE), AD patients with lower MMSE scores (using a cut-off of 20) exhibited significantly higher mean NLR (3.10 vs. 2.70, with a p-value for subgroup differences < 0.01).

**Conclusion:**

The NLR, which serves as a marker of peripheral inflammation, shows increased levels in individuals with AD and MCI compared to HC participants. Furthermore, our study indicates that NLR levels are significantly higher in AD than MCI. Additionally, our novel finding suggests significantly higher NLR levels among AD patients with more severe cognitive decline compared to AD patients with less severe cognitive decline. So, it can be concluded that the higher cognitive decline in humans is accompanied by higher NLR levels. Further longitudinal researches are needed to explore more details about the relationship between inflammation and dementia.

## 1. Introduction

In 2019, dementia accounted for 1.62 million fatalities worldwide, and the numbers are projected to increase significantly in the coming decades [1]. According to epidemiological data, Alzheimer’s disease (AD) is identified as the most prevalent form of dementia [2]. Currently, an estimated 6.5 million individuals aged 65 and above in the United States are affected by AD. Projections suggest that this number will nearly double, reaching approximately 13.8 million by 2060 [3]. This disease remains an incurable neurodegenerative condition that predominantly afflicts the elderly, resulting in a diminished quality of daily life, disability, and eventual mortality [4]. Typically, patients progress through an intermediate stage known as mild cognitive impairment (MCI) before receiving a formal AD diagnosis [5]. This intermediate stage, introduced in 1999, represents a phase of transition between typical cognitive function and AD. It indicates a population that is vulnerable to the development of AD [6].

In order to give a brief overview of AD mechanisms, it is important to acknowledge the key pathological changes that are associated with this condition. AD is distinguished by the presence of extracellular amyloid-β (Aβ) peptides, which give rise to neuritic plaques, as well as the build-up of intracellular hyperphosphorylated tau (p-tau) proteins, known as neurofibrillary tangles. These pathological features constitute the primary neuropathological standards for diagnosing AD [7]. It is worth noting that the formation of neurofibrillary tangles and Aβ plaques has been linked to inflammation and immune dysregulation [8].

The Neutrophil-to-Lymphocyte Ratio (NLR) is a clinical indicator of peripheral inflammation that has been extensively studied and is easily accessible. It is a straightforward calculation that indicates the equilibrium between the innate (neutrophils) and adaptive (lymphocytes) immune responses in different diseases and situations [9, 10]. Clinical investigations have explored the utility of NLR across a spectrum of diseases and conditions, including Parkinson’s disease (PD), amyotrophic lateral sclerosis, multiple sclerosis, and various cancers, demonstrating its predictive value [10–13]. Nearly a decade ago, researchers first reported higher NLR values in patients with AD [14], and this finding was subsequently supported by studies employing longitudinal data and repeated measurements over time [15].

The main goal of this systematic review and meta-analysis is to comprehensively evaluate the existing body of research regarding the NLR in the context of AD and MCI. Specifically, our objective is to examine the disparities in NLR levels among AD patients, individuals diagnosed with MCI, and healthy control (HC) participants. Additionally, we aim to explore the possible association between dementia severity, as measured by Mini-Mental State Examination (MMSE) scores, and NLR levels. By synthesizing and analyzing available data, our meta-analysis seeks to improve our understanding of the role of peripheral inflammation, as measured by NLR, in the onset and progression of AD and MCI. These findings would have implications for prevention, early detection, risk assessment, and developing of novel therapeutic approaches for these challenging cognitive disorders.

## 2. Method

In this investigation, we conducted a comprehensive systematic review and a subsequent meta-analysis, carefully adhering to the established guidelines outlined by the Preferred Reporting Items for Systematic Review and Meta-Analyses (PRISMA) checklist [16].

### 2.1. Literature search

The essential data for this systematic review and subsequent meta-analysis was assembled by conducting a comprehensive online search encompassing prominent databases, including MEDLINE, Scopus, EMBASE, and Web of Science, up to October 1st, 2023. The syntax for MEDLINE was: (“Alzheimer Disease” [mh] OR “Cognitive Dysfunction” [mh] OR “Dementia” [mh] OR Alzheimer [tiab] OR Cognitive Dysfunction [tiab] OR Cognitive Impairment [tiab] OR Cognitive Disorder [tiab] OR Mild Cognitive Impairment [tiab] OR Cognitive Decline [tiab] OR Dementia [tiab] OR Amentia [tiab]) AND (neutrophil lymphocyte ratio [tiab] OR neutrophil to lymphocyte ratio [tiab] OR neutrophil/lymphocyte ratio [tiab] OR NLR [tiab]); and we modified it for other databases. The reference lists of all the papers that were found were also assessed to guarantee the inclusion of all relevant studies. The search strategy for all databases is available in the S1 File.

### 2.2. Eligibility criteria

In order to maintain the precision and relevance of our systematic review, strict criteria were established for selecting primary studies. Eligible studies included peer-reviewed publications with a cross-sectional, case-control, or cohort design that evaluated the NLR in 1) patients with AD or MCI, and 2) HC participants, while providing mean and standard deviation (SD) data of NLR in each group.

Conversely, case reports and case series, clinical trials, animal studies, preprinted articles, review articles, studies that assessed NLR solely in AD/MCI patients, and studies that did not present NLR mean (SD) values for each group separately, were excluded. Furthermore, studies investigating NLR in forms of dementia or cognitive impairment other than AD/MCI were also excluded from our analysis.

### 2.3. Study selection

To ensure the accuracy and comprehensiveness of our systematic review, we adhered to a stringent and standardized protocol for screening and data collection. Two independent reviewers thoroughly assessed the titles and abstracts of all potentially relevant studies based on our predetermined criteria. Subsequently, the same reviewers conducted a detailed examination of the full-text articles of the selected papers to make the final decision on their inclusion in the review. In cases where discrepancies arose between the reviewers, a third reviewer was consulted to facilitate resolution, ensuring the utmost rigor and reliability in our study selection process.

For each article included in our review, two authors independently extracted pertinent data, which encompassed the first author’s name, publication year, study design, country of origin, diagnostic criteria employed for AD/MCI, demographic characteristics of the case and control groups, MMSE scores for the specified groups, and the apolipoprotein-E4 (APOEε4) status of participants. Furthermore, the necessary data from each study were carefully gathered to facilitate the computation of the mean difference (MD) in NLR across different group comparisons, namely AD vs HC, MCI vs HC, and AD vs MCI.

The data extraction process was carried out with great attention to detail, focusing on precision and reliability to underpin the robustness of our findings.

### 2.4. Quality assessment

For the assessment of study quality in this analysis, the Newcastle-Ottawa Scale (NOS) [17] was employed, which is designed for evaluating observational studies. The NOS assesses crucial facets such as sample selection, comparability between case and control groups, and exposure assessment. Ratings on the NOS scale range from 0 to 9, with higher scores signifying superior study quality. Our categorization of studies was predicated on their star ratings: studies with 7–9 stars were deemed of the highest quality, those with 4–6 stars were categorized as having lower quality, and studies with fewer than four stars were considered the lowest quality.

To ensure an impartial evaluation, two independent authors conducted the quality assessment of the included studies utilizing the NOS. Distinct checklists tailored to the particular study design were employed to ensure a comprehensive and accurate appraisal of each study’s quality. This rigorous approach maintained the integrity and objectivity of our quality assessment process.

### 2.5. Statistical analysis

For all our statistical analyses, R version 4.2.3 software was used. The MD of NLR was computed to enable meaningful comparisons of NLR between groups, incorporating a 95% confidence interval (CI) to elucidate the degree of certainty associated with our findings. Additionally, the pooled mean of NLR levels was calculated for each group.

To investigate whether the severity of AD and MCI impacted our results, a subgroup analysis was conducted for the pooled mean analysis based on MMSE scores. This analysis was contingent upon the availability of sufficient MMSE data within the meta-analysis. The cut-off for MMSE was set so that studies were divided into subgroups with nearly equal numbers of studies. In order to validate our results, the age of participants was checked as a potential confounding factor that may have affected our analyses using meta-regression.

For determining statistical significance, results with a two-tailed p-value less than 0.05 were considered. To assess the heterogeneity among the included studies, both the I2 statistic and the Cochrane Q statistic were employed. Given substantial heterogeneity in the data, a random-effects model was chosen for all analytical procedures. This approach accounted for the inherent variability among studies and ensured a robust analysis of our findings.

## 3. Result

### 3.1. Study selection

**Fig 1:**
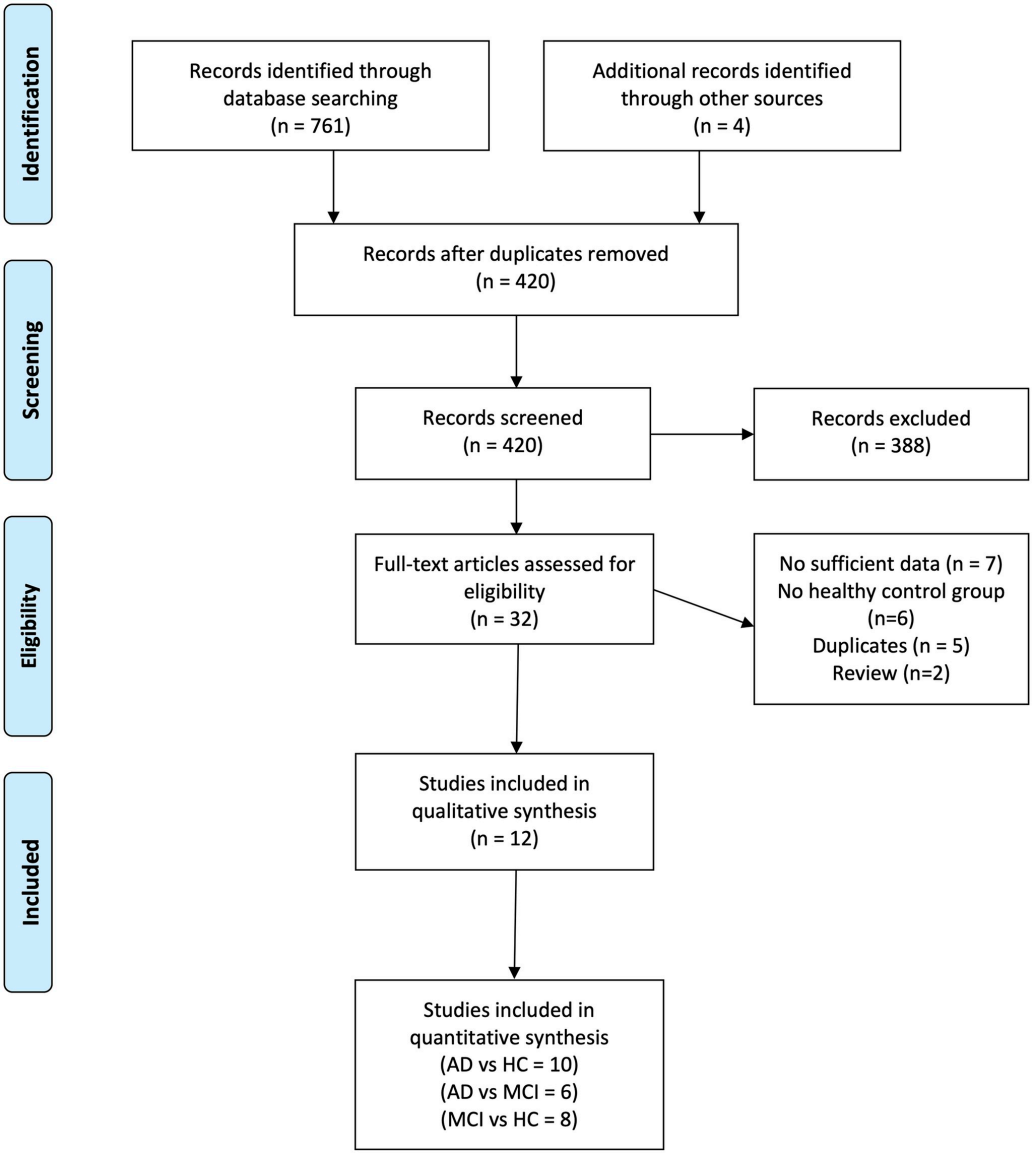
PRISMA diagram.

Following the elimination of duplicate records, our comprehensive search yielded 420 articles (as illustrated in Fig 1). A preliminary screening based on titles and abstracts led to the exclusion of 356 studies from this initial pool of articles. Subsequently, we conducted a meticulous review of the full texts of the remaining 64 articles. Ultimately, 12 studies aligned with our predefined inclusion criteria and were thus incorporated into our systematic review [14, 15, 18–27]. For a detailed assessment of the risk of bias associated with each study, please refer to Table 1, which provides detailed insights into the quality of the included research.

**Table 1:** Study characteristics.

Author	Year	Country	Study design	Disease	Diagnostic criteria	Number of subjects	Age	F/M	MMSE scores	APOE ε4
**An** [27]	2019	China	Case-Control	MCI		MCI: 186 HC: 153	MCI: 73.1 HC: 71.19	MCI: 108/78 HC: 81/72	MCI: 20.38 HC: 23.96	
**Dong** [18]	2019	China	Cross-Sectional	AD and MCI	NINCDS-ADRDA	AD: 56 MCI: 57 HC: 59	AD: 69.04 MCI: 70.67 HC: 68.12	AD: 33/23 MCI: 27/30 HC: 35/24		
**Evlice** [19]	2023	Turkey	Cross-Sectional	AD	NINCDS-ADRDA	AD: 132 HC: 38	AD: 70.83 HC: 71.09	AD: 69/63 HC: 25/13	AD: 19.39 HC: 28.04	
**Giannelli** [20]	2023	Italy	Cohort	MCI		MCI: 51 HC: 45		MCI: 30/21 HC: 29/16		
**Hou** [21]	2022	China	Cross-Sectional	AD and MCI		AD: 196 MCI: 620 HC: 291	AD: 74.35 MCI: 72.44 HC: 74.31	AD: 83/113 MCI: 257/363 HC: 146/145	AD: 23.24 MCI: 27.89 HC: 29.02	AD: 148 MCI: 307 HC: 76
**Kalelioglu** [22]	2017	Turkey	Cross-Sectional	AD and MCI	DSM-IV	AD: 31 MCI: 30 HC: 31		AD: 20/11 MCI: 19/11 HC: 20/11		
**Kara** [23]	2021	Turkey	Cross-Sectional	AD	DSM-IV	AD: 94 HC: 61	AD: 74.2 HC: 65.7	AD: 54/40 HC: 30/31		
**Kuyumcu** [14]	2012	Turkey	Cross-Sectional	AD	NINCDS-ADRDA and DSM-IV	AD: 241 HC: 175	AD: 76.53 HC: 71.95	AD: 165/76 HC: 78/97	AD: 18.32 HC: 27.08	
**Mehta** [24]	2023	USA	Cross-Sectional	AD and MCI		AD: 301 MCI: 838 HC: 405	AD: 75 MCI: 73 HC: 75	AD: 134/167 MCI: 343/495 HC: 201/204		AD: 205 MCI: 411 HC: 109
**Ohtani** [25]	2018	Japan	Cross-Sectional	AD and MCI	DSM-V and ICD-10	AD: 20 MCI: 17 HC: 17	AD: 76.5 MCI: 76.6 HC: 73.8	AD: 14/6 MCI: 11/6 HC: 12/5	AD: 20.4 MCI: 25.5 HC: 29.5	
**Rembach** [15]	2014	Australia	Cohort	AD and MCI	AD: NINCDS-ADRDA MCI: Peterson’s	AD: 205 MCI: 130 HC: 759	AD: 78.99 MCI: 76.25 HC: 70.57	AD: 125/80 MCI: 74/56 HC: 436/323	AD: 20 MCI: 26.5 HC: 29	AD: 127 MCI: 65 HC: 207
**Schröder** [26]	2022	Germany	Cohort	AD	ICD-10	AD: 33 HC: 30	AD: 76 HC: 76	AD: 26/7 HC: 13/7	AD: 17 HC: 28	

F/M: female/male, MMSE: Mini-Mental State Examination, APOEε4: apolipoprotein-E4, AD: Alzheimer’s disease, MCI: mild cognitive impairment, HC: healthy control, NINCDS-ADRDA: the National Institute of Neurological and Communicative Disorders and Stroke and the Alzheimer’s Disease and Related Disorders Association, DSM: Diagnostic and Statistical Manual of Mental Disorders, ICD: International Classification of Diseases.

### 3.2. Demographic characteristics

Collectively, our study encompassed a cohort of 1,309 individuals diagnosed with AD with a mean age of 75.13, 1,929 individuals with MCI with a mean age of 73.01, and 2,064 HC participants with a mean age of 72.09 across the 12 selected studies. The average age of the patients ranged from 65.7 to 78.99 years old.

Of the 12 studies, four originated from Turkey [14, 19, 22, 23], three from China [18, 21, 27], and one each from the USA and Canada [24], Italy [20], Germany [26], Australia [15], and Japan [25]. This international diversity underscores the global relevance and scope of our investigation.

### 3.3. Quality assessment

Most of the studies had high quality based on NOS. The detailed rating is available in Table 2.

**Table 2:** Quality assessment using NOS.

Author	Selection	Comparability	Exposure/Outcome	Overall
**An** [27]	3	2	2	7
**Dong** [18]	3	1	2	6
**Evlice** [19]	4	2	2	8
**Giannelli** [20]	3	1	2	6
**Hou** [21]	4	2	3	9
**Kalelioglu** [22]	3	2	2	7
**Kara** [23]	4	2	3	9
**Kuyumcu** [14]	2	1	2	5
**Mehta** [24]	4	2	3	9
**Ohtani** [25]	4	1	3	8
**Rembach** [15]	4	2	3	9
**Schröder** [26]	3	2	3	8

### 3.4. NLR Levels

The pooled mean of NLR levels in each group is:

- AD: 2.68 [2.49; 2.87]
- MCI: 2.42 [2.35; 2.49]
- HC: 2.06 [1.91; 2.21]

### 3.5. NLR mean difference

**Fig 2:**
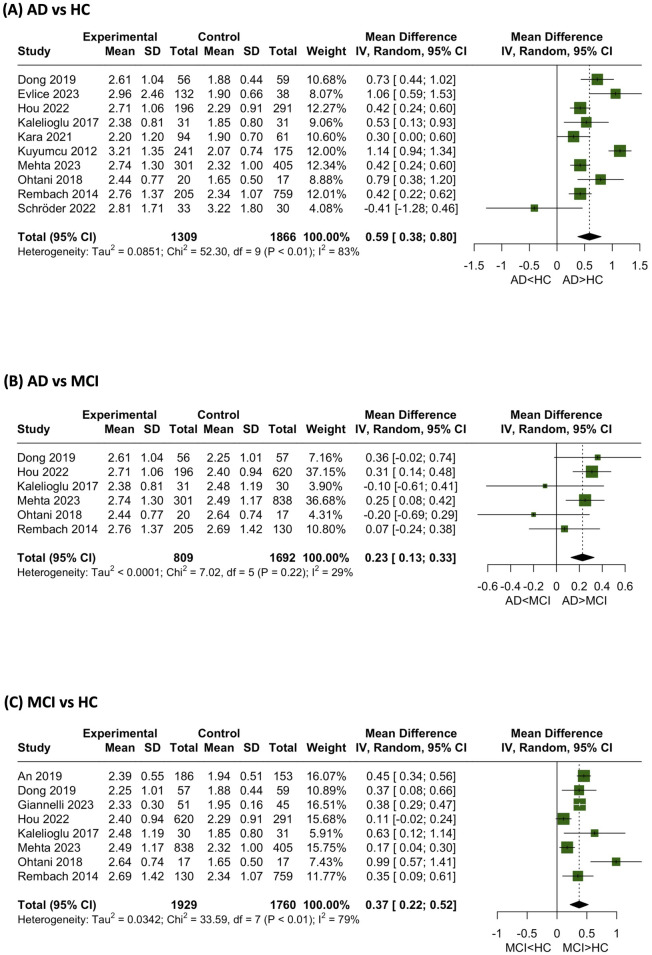
NLR mean differences.

In this investigation, NLR mean differences were conducted across three distinct sets: AD vs. HC, AD vs. MCI, and MCI vs. HC. Notably, statistically significant differences were observed in each of these comparisons (Fig 2):

- The mean NLR was notably higher in AD when compared to HC (MD = 0.59 [0.38; 0.80])
- Likewise, the mean NLR exhibited a higher level in AD when compared to MCI (MD = 0.23 [0.13; 0.33])
- Furthermore, the mean NLR was found to be elevated in MCI compared to HC (MD = 0.37 [0.22; 0.52])

Collectively, these findings lead to a compelling trend in the mean NLR across these three conditions, wherein the mean NLR levels follow the sequence AD > MCI > HC.

Meta-regression analysis showed that the age of participants did not have a significant effect on the above results.

### 3.6. Effect of disease severity

**Fig 3:**
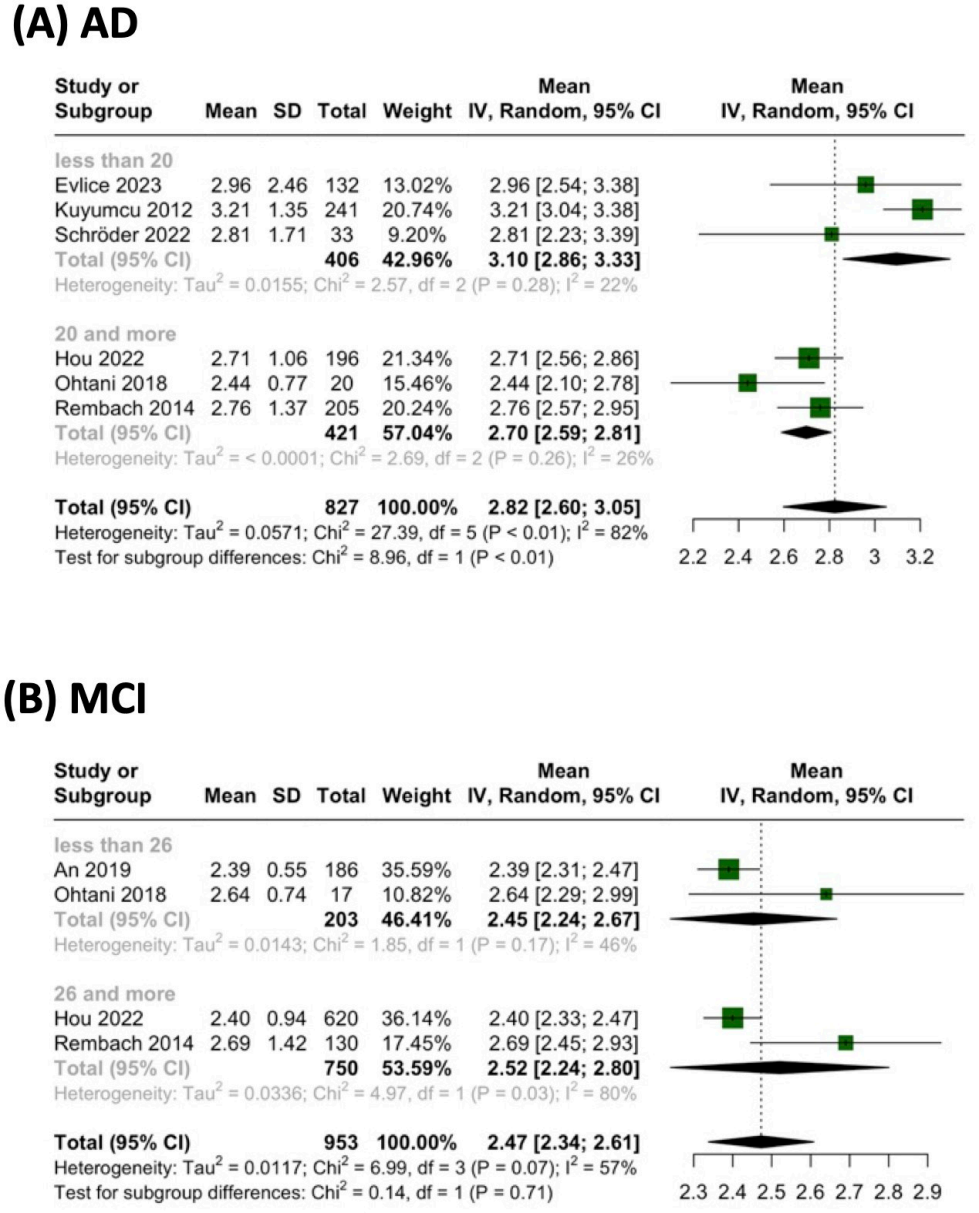
Subgroup meta-analysis based on MMSE score for the mean NLR levels in each group.

In our quest to discern any potential relationship between NLR and the severity of cognitive impairment, we undertook a subgroup meta-analysis based on MMSE scores (Fig 3). The results yielded insightful findings:

- In the subgroup meta-analysis based on MMSE, AD patients with lower MMSE scores (using a cut-off of 20) exhibited markedly higher mean NLR levels (3.10 vs. 2.70, with a p-value for subgroup differences < 0.01). This suggests a significant association between reduced cognitive function, as indicated by lower MMSE scores, and elevated mean NLR in AD patients.
- The subgroup meta-analysis based on MMSE revealed no significant differences between MCI patients with higher and lower MMSE scores (using a cut-off of 26). This implies that the association between NLR and cognitive impairment severity may not be as significant in MCI patients as it is in AD patients.

## 4. Discussion

### 4.1. Main findings

The main findings of our study were as follows:1) NLR exhibit a distinct pattern, with higher levels in AD compared to MCI, higher levels in MCI compared to HC, and NLR in AD is significantly higher than in HC, forming a hierarchy (NLR in AD > MCI > HC). 2) NLR levels in AD patients with lower MMSE scores are significantly higher than those with higher MMSE scores.

Despite significant research efforts, the exact causes and clinical characteristics of AD remain incompletely understood [28]. AD is primarily identified by the accumulation of extracellular Aβ peptides as plaques and the intracellular buildup of p-tau protein, which forms neurofibrillary tangles [7]. These plaques and tangles are believed to disrupt communication between nerve cells and vital cellular processes. This disruption leads to the death of nerve cells, resulting in memory loss, changes in personality, difficulties in daily activities, and other symptoms of AD [29–31].

Currently, there are no effective methods for preventing or treating the accumulation of Aβ or p-tau in patients with AD. Recent studies have shifted their focus towards exploring alternative factors that could prevent the formation of Aβ plaques and neurofibrillary tangles. These studies have revealed a connection between inflammation and irregularities in the immune system, which result in the formation of Aβ plaques and neurofibrillary tangles [8].

In the initial stages, the immune response triggers the activation of microglial cells, which are responsible for clearing these plaques, thus helping to protect against neurodegeneration [8]. However, prolonged inflammation leads to a loss of the microglial cells’ ability to clear these plaques [8, 32, 33]. Some earlier studies that employed translocator protein (TSPO) tracers to assess the activation of microglia in the brain identified correlations with the presence of Aβ accumulation as detected by positron emission tomography (PET) scans [34–36]. However, not all studies reported this connection [37].

As inflammation continues, it triggers the release of more inflammatory cytokines while simultaneously boosting the number of macrophages to address and combat the accumulation of plaque [38, 39]. For instance, studies have shown that interleukin-1 initiates a pathway mediated by protein kinase C, ultimately resulting in the expression of amyloid precursor protein (APP). APP is a larger precursor molecule generated by various cells, including neurons in the brain, cells in the blood vessels and blood, to a lesser degree, astrocytes. Subsequently, APP undergoes two breakdown processes facilitated by β-secretase (BACE1) and gamma-secretase, leading to the production of Aβ [40]. This process results in increased Aβ levels [41].

Inhibiting peripheral interleukin-1β has demonstrated a reduction in Aβ levels [42]. Additionally, interleukin-1β and tumor necrosis factor-alpha (TNF-α) can boost gamma-secretase activity, leading to increased APP cleavage into Aβ [43]. This creates a feedback loop that includes the accumulation of Aβ, activated microglia, and increased cytokines, ultimately leading to the expansion of neutrophils [44].

For example, heightened cytokines such as TNF-α have the capacity to stimulate neutrophil proliferation through a survival mechanism. This is facilitated by the secretion of interleukin-9 via a pathway that is dependent on NF-kB [45]. Neutrophils gathered near Aβ plaques contribute to neuronal damage by releasing neutrophil extracellular traps. This process is facilitated by the lymphocyte function-associated antigen 1 (LFA-1) integrin pathway [44, 46]. This further stimulates peripheral inflammation.

Previous research has indicated that when Aβ accumulates in the precuneus area of the brain, there is a change in the composition of lymphocytes. The transition from a "naïve" state to "memory" B cells causes a reduction in peripheral lymphocytes and an increase in lymphocytes within the central nervous system (CNS) [47, 48]. Moreover, neutrophils that are activated release inflammatory compounds and enzymes which obstruct lymphocyte activation in the bloodstream. For instance, these neutrophils can secrete proteases that cut off interleukin-6 and interleukin-2 receptors from the surface of T lymphocytes [49]. They also secrete arginase 1, an enzyme that depletes the arginine environment and reduces the activity of T cells [50]. By releasing reactive oxygen species (ROS) and altering cell adhesion mechanisms, neutrophils in an activated state can impair T lymphocyte function [51, 52]. These activated neutrophils have the capacity to reroute lymphocytes from peripheral regions towards the CNS. They achieve this by increasing the expression of matrix-metalloproteinase 9, which results in the disruption of the blood-brain barrier, thus permitting the migration of lymphocytes into the CNS [53, 54]. Consequently, a decrease in peripheral lymphocytes leads to an increase in the NLR.

In regards to the impact of longitudinal NLR increase on cognitive decline, it’s notable that the NLR correlates with more significant cognitive deterioration in all stages of the disease, but it does not appear to be linked to increased Aβ or tau accumulation after primary stages. This implies that the impact of activated microglia, Aβ plaques, and systemic inflammation is prominent during the initial phases of the disease. In advanced stages, systemic inflammation might contribute to mechanisms unrelated to Aβ or tau pathology [24].

In a 2019 study by Dong et al. in China, researchers investigated whether routine blood parameters could be used to diagnose AD. They examined data from AD patients, individuals with MCI, and HC participants, focusing on 17 different blood biomarkers. Eight of these biomarkers showed significant differences among the groups, with five being linked to both AD and MCI. Of particular interest were the inflammatory biomarkers, and the NLR stood out as a significant marker that could differentiate AD and MCI patients from HC participants [18].

Another study by Kalelioglu et al. in 2017 supported these findings [22]. However, according to a 2021 study conducted by Kara et al., no substantial variance in the NLR was observed when comparing AD patients to age-matched HC participants [23].

Hence, it is crucial to evaluate the differences in NLR levels solely as a diagnostic factor, while also accounting for the effects of age. In a 2012 study by Kuyumcu et al., elevated NLR levels in AD patients compared to HC participants were found to have high sensitivity, specificity, and predictive value for identifying AD. Multivariate regression analysis in this study confirmed NLR as an independent predictor for the presence of AD [14]. Furthermore, a 2023 study by Mehta et al. and some previous studies revealed that the elevated NLR observed was independent of various baseline variables in AD and MCI patients, including age [24, 46]—as confirmed in our study—, male sex [24], and APOEε4 carrier status [24]. This underscores the importance of NLR as a distinct and robust marker in their research [24]. Conversely, a 2014 cohort study conducted by Rembach et al. revealed that variations in NLR among AD and MCI patients were influenced by specific factors, such as age, sex, and APOEε4 status. Initially, in this study, the cross-sectional analysis, before adjustments for age, sex, and APOEε4 status, indicated significant differences in NLR between AD patients and HC participants. However, after adjusting for these factors, no significant elevation in NLR levels was observed. This suggests that these elements, rather than the disease process itself, accounted for the observed changes in NLR. Additionally, longitudinal analyses conducted to assess the role of AD in increasing NLR over time demonstrated that the rise in NLR levels was significantly different before adjustments for age, sex, and APOEε4 status. Yet, after adjusting for these factors, NLR levels did not show significant differences between AD patients and HC participants. Consequently, these factors significantly influenced NLR differences, thereby limiting the utility of NLR for diagnosis or prognosis [15].

Understanding the differences in NLR levels between AD and MCI patients is crucial for evaluating treatments that aim to address peripheral inflammation and potentially prevent the progression from MCI to AD. While Dong et al.’s study indicated an observable variation in NLR differences between AD and MCI patients, it did not reach statistical significance [18]. In contrast, our research demonstrates elevated NLR levels in AD patients compared to those with MCI. The observed significant difference in NLR levels among AD and MCI patients might be attributed to the influence of a longitudinal rise in NLR over time, impacting cognitive decline, as discussed in Mehta et al.’s study [24]. This increase in NLR levels appears to align with the severity of cognitive deterioration. This reasoning may justify another significant finding we’ve previously mentioned. It has been revealed that NLR levels in AD patients with lower MMSE scores are significantly higher than those with higher MMSE scores. However, MCI patients with lower MMSE scores do not exhibit significantly higher levels than those with higher MMSE scores. The MMSE is a widely recognized screening tool for assessing cognitive impairment and dementia [55] and serves as an indicator of the severity of cognitive impairment [56]. Our findings suggest that AD patients with higher disease severity tend to have higher NLR levels. In line with this, a prior study demonstrated an association between NLR and longitudinal changes in the AD Assessment Scale Cognitive Subscale (ADAS-Cog) score, a tool for assessing cognitive function. Regarding the Impact of Longitudinal NLR Increase on Cognitive Decline, it is noteworthy that while NLR correlates with more significant cognitive deterioration across all disease stages, it does not seem connected to increased Aβ or tau accumulation after the initial stages. This suggests that during the early phases of the disease, the impact of activated microglia, Aβ plaques, and systemic inflammation is notable. In later stages, systemic inflammation might contribute to mechanisms unrelated to Aβ or tau pathology [24]. To the best of our knowledge, our study is the first to report the association of NLR levels with cognitive decline in AD patients based on MMSE. With these findings, further essential research can delve deeper into this relationship, providing a definitive explanation and mechanisms for these observed differences.

### 4.2. Clinical relevance

In recent years, the connection between AD and inflammation has drawn significant attention. Our findings suggest a relationship between NLR levels and the severity of cognitive decline. Furthermore, according to previous studies, elevated NLR levels serve as a predictor for postoperative complications, peri-procedural and post-procedural mortality, irrespective of the surgery type [57–59], as well as cardiovascular events [60], and poor prognosis for stroke patients [61]. These conditions are more prevalent in the elderly, similar to AD. NLR serves as a crucial marker across various disease conditions, such as heart disease, stroke, cancer, and PD, providing insights into their pathophysiology and clinical management. In cardiac disorders, studies have shown an association between elevated NLR levels and an increased risk of coronary artery disease, suggesting its potential as a predictive tool for myocardial damage and cardiac dysfunction [62, 63]. Dynamic changes in NLR could serve as early indicators of pathological states such as cancer, infection, and inflammation, emphasizing its role beyond cardiovascular health [10]. In cancer, NLR emerges as a significant marker for prognosis and treatment response prediction across various cancer types. Its role in risk assessment and therapeutic decision-making underscores its potential to enhance cancer management strategies [64, 65]. Elevated NLR levels have been linked to poor outcomes in both localized and metastatic cancer, indicating its prognostic significance across various cancer types [66, 67]. An NLR cut-off of ≥5 is commonly used to define an abnormal elevation in metastatic cancer, with higher NLR values associated with worse outcomes [66]. Studies have shown that high NLR values are linked to worse overall survival and cancer-specific survival, making it a valuable tool for risk assessment and predicting patient outcomes [65, 68]. NLR has also been explored in the context of treatment response prediction, where initial and post-treatment NLR levels are evaluated to assess their predictive value in disease progression and response to therapy [67].

Furthermore, elevated levels of NLR have been associated with poor outcomes in stroke patients. These outcomes include increased mortality, poor prognosis, and the occurrence of hemorrhagic transformation [61, 69]. Studies have shown that high NLR values are independently correlated with severe stroke and poor functional outcomes, particularly in acute ischemic stroke patients with intracranial atherosclerotic stenosis [69]. While NLR is primarily used for prognosis in stroke, ongoing research is exploring its potential role in prevention, early detection, and novel therapeutic strategies. In PD, the literature indicates that a higher NLR is associated with the severity of PD, suggesting its potential as a disease marker [11, 70]. Elevated NLR values have been observed in PD patients compared to healthy individuals, highlighting its diagnostic value and potential role in assessing disease presence [11]. Moreover, studies have shown that an increased NLR is highly correlated with the presence of PD, emphasizing the need for further research to explore the clinical benefits of this biomarker in PD management [11].

Overall, the dynamic nature of NLR and its associations with various disease conditions emphasize its promising utility in preventive measures, early detection, risk assessment, and the development of novel therapeutic approaches. These warrants continued investigation into its broader clinical applications. All of these emphasize the increasing importance of targeting inflammation. Targeting inflammation may hold promise as both a therapeutic and preventative approach for AD [71].

Current therapeutic strategies for AD encompass a range of mechanisms, including the removal of Aβ plaques, addressing the accumulation of tau proteins, modulating the function of apolipoprotein-E (ApoE), neuroprotection, and managing neuroinflammatory responses. Additionally, non-mechanism-based strategies focus on alleviating cognitive symptoms, preventing the onset of AD, making lifestyle adjustments, and managing risk factors [72]. It is crucial to emphasize that, as of now, there is no cure for AD, and the available treatments primarily aim to manage the symptoms. Therefore, a more practical approach lies in preventing the onset of AD, as this approach can significantly reduce the burden of the condition on affected individuals, their families, and healthcare systems.

While the levels of Aβ and p-tau proteins in CSF have demonstrated their worth as biomarkers for identifying elderly individuals at risk of dementia development [73], their use is limited due to the invasive and challenging nature of CSF sampling. Hence, there is a growing need for easily accessible screening markers, like those found in peripheral blood cell profiles, which could offer practical advantages in clinical practice. These markers have the potential to revolutionize the early detection of individuals susceptible to AD, allowing for timely interventions and personalized care.

### 4.3. Limitations of the study

This study has several limitations:

1. **Multifactorial Influence on NLR:** The NLR, as an indicator of peripheral inflammation, is influenced by a wide range of factors. These factors include age, sex, race, body mass index, marital status, alcohol consumption, physical activity, smoking history, infections, use of exogenous steroids, endogenous hormonal levels, active hematologic disorders, leukemia, cytotoxic chemotherapy, and the administration of granulocyte colony-stimulating factor (G-CSF).
2. **Geographical Bias:** A significant portion of the studies (seven out of twelve) included in our analysis were conducted in only two countries, suggesting a potential geographical bias. Additional research involving diverse racial populations is needed to enhance the generalizability of our findings.
3. **Limited Subgroup Analysis:** Due to the scarcity of available data, this meta-analysis was unable to explore more specific subgroups that could have provided more profound insights.
4. **Lack of Data on NLR and Mortality:** This study encountered limitations due to the lack of available data on the relationship between NLR and mortality in AD and MCI patients across the included studies. The absence of sufficient data prevented a comprehensive analysis of the potential association between NLR levels and mortality outcomes in these populations. Therefore, the impact of NLR on mortality in AD and MCI patients remains inadequately explored, highlighting a significant gap in the current literature. Future research addressing this aspect would contribute to a more comprehensive understanding of the prognostic significance of NLR in neurodegenerative diseases.
5. **MMSE Score Association**: There is currently insufficient data available to analyze the correlation between MMSE scores and NLR directly.
6. **Use of Cross-Sectional Data:** This meta-analysis and systematic review study utilized cross-sectional data of patients’ NLR due to the lack of longitudinal data. Therefore, further longitudinal studies are required to obtain more robust and confident results, specifically to investigate if elevated NLR at a younger age is associated with a higher risk of cognitive decline.

## 5. Conclusion

Our research contributes to the expanding body of evidence that reinforces the notion that the AD development is linked to alterations in peripheral inflammatory and immune profiles. The NLR, which serves as a marker of peripheral inflammation, exhibits increased levels in individuals with both AD and MCI when compared to HC participants. Additionally, this study reveals that NLR levels are significantly higher in AD compared to MCI. Moreover, our novel finding suggests a significant elevation in NLR levels among AD patients with lower MMSE scores compared to those with higher MMSE scores. In summary, our research provides valuable insights into the association of NLR and the pathogenesis of AD. Nevertheless, future studies with larger sample sizes need to continue investigating these associations and their clinical implications.

## Supporting information

S1 File Our search strategy for searching in databases. (DOCX)

S1 Checklist PRISMA 2020 checklist. (DOCX)

## References

[1] Nichols E. Estimating the global mortality from Alzheimers disease and other dementias: a new method and results from the Global Burden of Disease Study 2019 2019.

[2] Garre-Olmo J. Epidemiología de la enfermedad de Alzheimer y otras demencias. Rev Neurol. 2018;66(11):377–86. 29790571

[3] Gaugler J, James B, Johnson T, Reimer J, Solis M, Weuve J, et al. 2022 Alzheimer’s disease facts and figures. Alzheimers & Dementia. 2022;18(4):700–89. 10.1002/alz.12638 35289055

[4] Association As. 2015 Alzheimer’s disease facts and figures. Alzheimer’s & Dementia. 2015;11(3):332–84. doi: 10.1016/j.jalz.2015.02.003 25984581

[5] Petersen RC, Caracciolo B, Brayne C, Gauthier S, Jelic V, Fratiglioni L. Mild cognitive impairment: a concept in evolution. Journal of internal medicine. 2014;275(3):214–28. doi: 10.1111/joim.12190 24605806 PMC3967548

[6] Bradfield NI. Mild cognitive impairment: diagnosis and subtypes. Clinical EEG and Neuroscience. 2023;54(1):4–11. doi: 10.1177/15500594211042708 34549629

[7] Winblad B, Amouyel P, Andrieu S, Ballard C, Brayne C, Brodaty H, et al. Defeating Alzheimer’s disease and other dementias: a priority for European science and society. The Lancet Neurology. 2016;15(5):455–532. doi: 10.1016/S1474-4422(16)00062-4 26987701

[8] Sayed A, Bahbah EI, Kamel S, Barreto GE, Ashraf GM, Elfil M. The neutrophil-to-lymphocyte ratio in Alzheimer’s disease: Current understanding and potential applications. Journal of Neuroimmunology. 2020;349:577398. doi: 10.1016/j.jneuroim.2020.577398 32977249

[9] Buonacera A, Stancanelli B, Colaci M, Malatino L. Neutrophil to lymphocyte ratio: an emerging marker of the relationships between the immune system and diseases. International journal of molecular sciences. 2022;23(7):3636. doi: 10.3390/ijms23073636 35408994 PMC8998851

[10] Zahorec R. Neutrophil-to-lymphocyte ratio, past, present and future perspectives. Bratisl Lek Listy. 2021;122(7):474–88. doi: 10.4149/BLL_2021_078 34161115

[11] Hosseini S, Shafiabadi N, Khanzadeh M, Ghaedi A, Ghorbanzadeh R, Azarhomayoun A, et al. Neutrophil to lymphocyte ratio in Parkinson’s disease: A systematic review and meta-analysis. BMC neurology. 2023;23(1):333. doi: 10.1186/s12883-023-03380-7 37735638 PMC10512499

[12] Choi S-J, Hong Y-H, Kim S-M, Shin J-Y, Suh YJ, Sung J-J. High neutrophil-to-lymphocyte ratio predicts short survival duration in amyotrophic lateral sclerosis. Scientific reports. 2020;10(1):428. doi: 10.1038/s41598-019-57366-y 31949271 PMC6965090

[13] Hasselbalch I, Søndergaard H, Koch-Henriksen N, Olsson A, Ullum H, Sellebjerg F, et al. The neutrophil-to-lymphocyte ratio is associated with multiple sclerosis. Multiple Sclerosis Journal–Experimental, Translational and Clinical. 2018;4(4):2055217318813183. doi: 10.1177/2055217318813183 30515298 PMC6262498

[14] Kuyumcu ME, Yesil Y, Oztürk ZA, Kizilarslanoğlu C, Etgül S, Halil M, et al. The evaluation of neutrophil-lymphocyte ratio in Alzheimer’s disease. Dementia and geriatric cognitive disorders. 2012;34(2):69–74. doi: 10.1159/000341583 22922667

[15] Rembach A, Watt AD, Wilson WJ, Rainey-Smith S, Ellis KA, Rowe CC, et al. An increased neutrophil–lymphocyte ratio in Alzheimer’s disease is a function of age and is weakly correlated with neocortical amyloid accumulation. Journal of neuroimmunology. 2014;273(1–2):65–71. doi: 10.1016/j.jneuroim.2014.05.005 24907904

[16] Moher D, Shamseer L, Clarke M, Ghersi D, Liberati A, Petticrew M, et al. Preferred reporting items for systematic review and meta-analysis protocols (PRISMA-P) 2015 statement. Systematic reviews. 2015;4:1–9. doi: 10.1186/2046-4053-4-1 25554246 PMC4320440

[17] Wells GA, Shea B, O’Connell D, Peterson J, Welch V, Losos M, et al. The Newcastle-Ottawa Scale (NOS) for assessing the quality of nonrandomised studies in meta-analyses. 2000.

[18] Dong X, Nao J, Shi J, Zheng D. Predictive value of routine peripheral blood biomarkers in Alzheimer’s disease. Frontiers in aging neuroscience. 2019;11:332. doi: 10.3389/fnagi.2019.00332 31866854 PMC6906180

[19] Evlice A, Sanli ZS, Boz PB. The importance of Vitamin-D and Neutrophil-Lymphocyte Ratio for Alzheimer’s Disease. Pakistan Journal of Medical Sciences. 2023;39(3):799. doi: 10.12669/pjms.39.3.7024 37250565 PMC10214823

[20] Giannelli R, Canale P, Del Carratore R, Falleni A, Bernardeschi M, Forini F, et al. Ultrastructural and Molecular Investigation on Peripheral Leukocytes in Alzheimer’s Disease Patients. International Journal of Molecular Sciences. 2023;24(9):7909. doi: 10.3390/ijms24097909 37175616 PMC10178539

[21] Hou J-H, Ou Y-N, Xu W, Zhang P-F, Tan L, Yu J-T, et al. Association of peripheral immunity with cognition, neuroimaging, and Alzheimer’s pathology. Alzheimer’s Research & Therapy. 2022;14(1):29. doi: 10.1186/s13195-022-00968-y 35139899 PMC8830026

[22] Kalelioglu T, Yuruyen M, Gultekin G, Yavuzer H, Ozturk Y, Kurt M, et al. The neutrophil and platelet to lymphocyte ratios in people with subjective, mild cognitive impairment and early Alzheimer’s disease. European Psychiatry. 2017;41(S1):S655–S. 10.1111/psyg.12260 28386987

[23] Kara SP, Altunan B, Unal A. Investigation of the peripheral inflammation (neutrophil–lymphocyte ratio) in two neurodegenerative diseases of the central nervous system. Neurological Sciences. 2022:1–9. doi: 10.1007/s10072-021-05507-5 34331157 PMC8324446

[24] Mehta NH, Zhou L, Li Y, McIntire LB, Nordvig A, Butler T, et al. Peripheral immune cell imbalance is associated with cortical beta-amyloid deposition and longitudinal cognitive decline. Scientific Reports. 2023;13(1):8847. doi: 10.1038/s41598-023-34012-2 37258519 PMC10232445

[25] Ohtani R, Nirengi S, Nakamura M, Murase N, Sainouchi M, Kuwata Y, et al. High-density lipoprotein subclasses and mild cognitive Impairment: Study of outcome and aPolipoproteins in dementia (STOP-dementia). Journal of Alzheimer’s Disease. 2018;66(1):289–96. 10.3233/JAD-180135 30248050

[26] Schröder S, Heck J, Groh A, Frieling H, Bleich S, Kahl KG, et al. White blood cell and platelet counts are not suitable as biomarkers in the differential diagnostics of dementia. Brain Sciences. 2022;12(11):1424. doi: 10.3390/brainsci12111424 36358351 PMC9688654

[27] An P, Zhou X, Du Y, Zhao J, Song A, Liu H, et al. Association of neutrophil-lymphocyte ratio with mild cognitive impairment in elderly Chinese adults: a case-control study. Current Alzheimer Research. 2019;16(14):1309–15. doi: 10.2174/1567205017666200103110521 31902361

[28] Long JM, Holtzman DM. Alzheimer disease: an update on pathobiology and treatment strategies. Cell. 2019;179(2):312–39. doi: 10.1016/j.cell.2019.09.001 31564456 PMC6778042

[29] Guillozet AL, Weintraub S, Mash DC, Mesulam MM. Neurofibrillary tangles, amyloid, and memory in aging and mild cognitive impairment. Archives of neurology. 2003;60(5):729–36. doi: 10.1001/archneur.60.5.729 12756137

[30] Alzheimer’s Disease Fact Sheet: National Institutes of Health; 2023 [https://www.nia.nih.gov/health/alzheimers-and-dementia/alzheimers-disease-fact-sheet.

[31] Perl DP. Neuropathology of Alzheimer’s disease. Mount Sinai Journal of Medicine: A Journal of Translational and Personalized Medicine: A Journal of Translational and Personalized Medicine. 2010;77(1):32–42. doi: 10.1002/msj.20157 20101720 PMC2918894

[32] Wyss-Coray T, Yan F, Lin AH-T, Lambris JD, Alexander JJ, Quigg RJ, et al. Prominent neurodegeneration and increased plaque formation in complement-inhibited Alzheimer’s mice. Proceedings of the National Academy of Sciences. 2002;99(16):10837–42. doi: 10.1073/pnas.162350199 12119423 PMC125059

[33] Simard AR, Soulet D, Gowing G, Julien J-P, Rivest S. Bone marrow-derived microglia play a critical role in restricting senile plaque formation in Alzheimer’s disease. Neuron. 2006;49(4):489–502. doi: 10.1016/j.neuron.2006.01.022 16476660

[34] Okello A, Edison P, Archer H, Turkheimer F, Kennedy J, Bullock R, et al. Microglial activation and amyloid deposition in mild cognitive impairment: a PET study. Neurology. 2009;72(1):56–62. doi: 10.1212/01.wnl.0000338622.27876.0d 19122031 PMC2817573

[35] McGeer PL, Itagaki S, Tago H, McGeer EG. Reactive microglia in patients with senile dementia of the Alzheimer type are positive for the histocompatibility glycoprotein HLA-DR. Neuroscience letters. 1987;79(1–2):195–200. doi: 10.1016/0304-3940(87)90696-3 3670729

[36] Zheng C, Zhou X-W, Wang J-Z. The dual roles of cytokines in Alzheimer’s disease: update on interleukins, TNF-α, TGF-β and IFN-γ. Translational neurodegeneration. 2016;5(1):1–15. 27054030 10.1186/s40035-016-0054-4 PMC4822284

[37] Hansen DV, Hanson JE, Sheng M. Microglia in Alzheimer’s disease. Journal of Cell Biology. 2018;217(2):459–72. doi: 10.1083/jcb.201709069 29196460 PMC5800817

[38] Stalder AK, Ermini F, Bondolfi L, Krenger W, Burbach GJ, Deller T, et al. Invasion of hematopoietic cells into the brain of amyloid precursor protein transgenic mice. Journal of Neuroscience. 2005;25(48):11125–32. doi: 10.1523/JNEUROSCI.2545-05.2005 16319312 PMC6725647

[39] Krabbe G, Halle A, Matyash V, Rinnenthal JL, Eom GD, Bernhardt U, et al. Functional impairment of microglia coincides with Beta-amyloid deposition in mice with Alzheimer-like pathology. PloS one. 2013;8(4):e60921. doi: 10.1371/journal.pone.0060921 23577177 PMC3620049

[40] Hampel H, Hardy J, Blennow K, Chen C, Perry G, Kim SH, et al. The amyloid-β pathway in Alzheimer’s disease. Molecular psychiatry. 2021;26(10):5481–503. 34456336 10.1038/s41380-021-01249-0 PMC8758495

[41] Goldgaber D, Harris HW, Hla T, Maciag T, Donnelly RJ, Jacobsen JS, et al. Interleukin 1 regulates synthesis of amyloid beta-protein precursor mRNA in human endothelial cells. Proceedings of the National Academy of Sciences. 1989;86(19):7606–10. doi: 10.1073/pnas.86.19.7606 2508093 PMC298115

[42] Kitazawa M, Cheng D, Tsukamoto MR, Koike MA, Wes PD, Vasilevko V, et al. Blocking IL-1 signaling rescues cognition, attenuates tau pathology, and restores neuronal β-catenin pathway function in an Alzheimer’s disease model. The Journal of Immunology. 2011;187(12):6539–49. 22095718 10.4049/jimmunol.1100620 PMC4072218

[43] Liao Y-F, Wang B-J, Cheng H-T, Kuo L-H, Wolfe MS. Tumor necrosis factor-α, interleukin-1β, and interferon-γ stimulate γ-secretase-mediated cleavage of amyloid precursor protein through a JNK-dependent MAPK pathway. Journal of Biological Chemistry. 2004;279(47):49523–32. 15347683 10.1074/jbc.M402034200

[44] Swardfager W, Lanctôt K, Rothenburg L, Wong A, Cappell J, Herrmann N. A meta-analysis of cytokines in Alzheimer’s disease. Biological psychiatry. 2010;68(10):930–41. doi: 10.1016/j.biopsych.2010.06.012 20692646

[45] AS C. The survival effect of TNF-α in human neutrophils is mediated via NF-κB-dependent IL-8 release. Eur J Immunol. 2004;34:1733–43. 15162444 10.1002/eji.200425091

[46] Li J, Chen Q, Luo X, Hong J, Pan K, Lin X, et al. Neutrophil‐to‐lymphocyte ratio positively correlates to age in healthy population. Journal of clinical laboratory analysis. 2015;29(6):437–43. doi: 10.1002/jcla.21791 25277347 PMC6807196

[47] Richartz-Salzburger E, Batra A, Stransky E, Laske C, Köhler N, Bartels M, et al. Altered lymphocyte distribution in Alzheimer’s disease. Journal of psychiatric research. 2007;41(1–2):174–8. doi: 10.1016/j.jpsychires.2006.01.010 16516234

[48] Stowe AM, Ireland SJ, Ortega SB, Chen D, Huebinger RM, Tarumi T, et al. Adaptive lymphocyte profiles correlate to brain Aβ burden in patients with mild cognitive impairment. Journal of neuroinflammation. 2017;14(1):1–11. 28750671 10.1186/s12974-017-0910-x PMC5530920

[49] Bank U, Reinhold D, Schneemilch C, Kunz D, Synowitz H-J, Ansorge S. Selective proteolytic cleavage of IL-2 receptor and IL-6 receptor ligand binding chains by neutrophil-derived serine proteases at foci of inflammation. Journal of interferon & cytokine research. 1999;19(11):1277–87. doi: 10.1089/107999099312957 10574621

[50] Munder M, Schneider H, Luckner C, Giese T, Langhans C-D, Fuentes JM, et al. Suppression of T-cell functions by human granulocyte arginase. Blood. 2006;108(5):1627–34. doi: 10.1182/blood-2006-11-010389 16709924

[51] Aarts CE, Hiemstra IH, Béguin EP, Hoogendijk AJ, Bouchmal S, van Houdt M, et al. Activated neutrophils exert myeloid-derived suppressor cell activity damaging T cells beyond repair. Blood advances. 2019;3(22):3562–74. doi: 10.1182/bloodadvances.2019031609 31738831 PMC6880908

[52] El-Hag A, Clark R. Immunosuppression by activated human neutrophils. Dependence on the myeloperoxidase system. Journal of immunology (Baltimore, Md: 1950). 1987;139(7):2406–13. 2821114

[53] Pun PB, Lu J, Moochhala S. Involvement of ROS in BBB dysfunction. Free radical research. 2009;43(4):348–64. doi: 10.1080/10715760902751902 19241241

[54] Dong Y, Lagarde J, Xicota L, Corne H, Chantran Y, Chaigneau T, et al. Neutrophil hyperactivation correlates with Alzheimer’s disease progression. Annals of neurology. 2018;83(2):387–405. doi: 10.1002/ana.25159 29369398

[55] Folstein MF, Folstein SE, McHugh PR. “Mini-mental state”: a practical method for grading the cognitive state of patients for the clinician. Journal of psychiatric research. 1975;12(3):189–98. 1202204 10.1016/0022-3956(75)90026-6

[56] Henneges C, Reed C, Chen Y-F, Dell’Agnello G, Lebrec J. Describing the sequence of cognitive decline in Alzheimer’s disease patients: results from an observational study. Journal of Alzheimer’s Disease. 2016;52(3):1065–80. doi: 10.3233/JAD-150852 27079700 PMC4927893

[57] Josse J, Cleghorn M, Ramji K, Jiang H, Elnahas A, Jackson T, et al. The neutrophil‐to‐lymphocyte ratio predicts major perioperative complications in patients undergoing colorectal surgery. Colorectal Disease. 2016;18(7):O236–O42. doi: 10.1111/codi.13373 27154050

[58] Silberman S, Abu-Yunis U, Tauber R, Shavit L, Grenader T, Fink D, et al. Neutrophil-lymphocyte ratio: prognostic impact in heart surgery. Early outcomes and late survival. The Annals of thoracic surgery. 2018;105(2):581–6. doi: 10.1016/j.athoracsur.2017.07.033 29132702

[59] Hajibandeh S, Hajibandeh S, Hobbs N, Mansour M. Neutrophil-to-lymphocyte ratio predicts acute appendicitis and distinguishes between complicated and uncomplicated appendicitis: A systematic review and meta-analysis. The American Journal of Surgery. 2020;219(1):154–63. doi: 10.1016/j.amjsurg.2019.04.018 31056211

[60] Shah N, Parikh V, Patel N, Patel N, Badheka A, Deshmukh A, et al. Neutrophil lymphocyte ratio significantly improves the Framingham risk score in prediction of coronary heart disease mortality: insights from the National Health and Nutrition Examination Survey-III. International journal of cardiology. 2014;171(3):390–7. doi: 10.1016/j.ijcard.2013.12.019 24388541

[61] Li W, Hou M, Ding Z, Liu X, Shao Y, Li X. Prognostic value of neutrophil-to-lymphocyte ratio in stroke: a systematic review and meta-analysis. Frontiers in Neurology. 2021;12:686983. doi: 10.3389/fneur.2021.686983 34630275 PMC8497704

[62] Wang Y, Zhuang Y, Lin C, Hong H, Chen F, Ke J. The neutrophil-to-lymphocyte ratio is associated with coronary heart disease risk in adults: A population-based study. Plos one. 2024;19(2):e0296838. doi: 10.1371/journal.pone.0296838 38349930 PMC10863873

[63] Chen C, Cong BL, Wang M, Abdullah M, Wang XL, Zhang YH, et al. Neutrophil to lymphocyte ratio as a predictor of myocardial damage and cardiac dysfunction in acute coronary syndrome patients. Integrative medicine research. 2018;7(2):192–9. doi: 10.1016/j.imr.2018.02.006 29984180 PMC6026362

[64] Faria SS, Fernandes PC Jr, Silva MJB, Lima VC, Fontes W, Freitas-Junior R, et al. The neutrophil-to-lymphocyte ratio: a narrative review. ecancermedicalscience. 2016;10. doi: 10.3332/ecancer.2016.702 28105073 PMC5221645

[65] Cupp MA, Cariolou M, Tzoulaki I, Aune D, Evangelou E, Berlanga-Taylor AJ. Neutrophil to lymphocyte ratio and cancer prognosis: an umbrella review of systematic reviews and meta-analyses of observational studies. BMC medicine. 2020;18:1–16. 33213430 10.1186/s12916-020-01817-1 PMC7678319

[66] Bartlett EK, Flynn JR, Panageas KS, Ferraro RA, Sta. Cruz JM, Postow MA, et al. High neutrophil‐to‐lymphocyte ratio (NLR) is associated with treatment failure and death in patients who have melanoma treated with PD‐1 inhibitor monotherapy. Cancer. 2020;126(1):76–85. doi: 10.1002/cncr.32506 31584709 PMC6906249

[67] Shin H-n, Kim J, Kim HJ. Neutrophil lymphocyte ratio (NLR) change after systemic treatment as a predictive factor of cancer specific survival in stage IV breast cancer. American Society of Clinical Oncology; 2015.

[68] Ma SJ, Yu H, Khan M, Gill J, Santhosh S, Chatterjee U, et al. Evaluation of optimal threshold of neutrophil-lymphocyte ratio and its association with survival outcomes among patients with head and neck cancer. JAMA Network Open. 2022;5(4):e227567–e. doi: 10.1001/jamanetworkopen.2022.7567 35426920 PMC9012962

[69] Ying Y, Yu F, Luo Y, Feng X, Liao D, Wei M, et al. Neutrophil-to-lymphocyte ratio as a predictive biomarker for stroke severity and short-term prognosis in acute ischemic stroke with intracranial atherosclerotic stenosis. Frontiers in neurology. 2021;12:705949. doi: 10.3389/fneur.2021.705949 34393983 PMC8360230

[70] Li F, Weng G, Deng B, Zhu S, Wang Q. The neutrophil-to-lymphocyte ratio, lymphocyte-to-monocyte ratio, and neutrophil-to-high-density-lipoprotein ratio are correlated with the severity of Parkinson’s disease. Frontiers in Neurology. 2024;15:1322228. doi: 10.3389/fneur.2024.1322228 38322584 PMC10844449

[71] Rather MA, Khan A, Alshahrani S, Rashid H, Qadri M, Rashid S, et al. Inflammation and Alzheimer’s disease: mechanisms and therapeutic implications by natural products. Mediators of Inflammation. 2021;2021:1–21. 10.1155/2021/9982954 PMC8352708 34381308

[72] Cao J, Hou J, Ping J, Cai D. Advances in developing novel therapeutic strategies for Alzheimer’s disease. Molecular neurodegeneration. 2018;13:1–20. 30541602 10.1186/s13024-018-0299-8 PMC6291983

[73] Langa KM, Levine DA. The diagnosis and management of mild cognitive impairment: a clinical review. Jama. 2014;312(23):2551–61. doi: 10.1001/jama.2014.13806 25514304 PMC4269302

