# Supplementary material for: Neutrophil to lymphocyte ratio in Alzheimer’s disease: A systematic review and meta-analysis
Source: PLoS One. 2024 Jun 25;19(6):e0305322. doi: 10.1371/journal.pone.0305322 (PMC11198755; doi:10.1371/journal.pone.0305322)
Supplement: S1 File — (DOCX) [file pone.0305322.s001.docx]

Date: 10/01/2023

PubMed=158

Embase=268

Scopus=157

WOS=178

Total: 761

Non-duplicate: 420

**Pubmed**

**“Alzheimer Disease” [mh] OR “Cognitive Dysfunction” [mh] OR “Dementia” [mh] OR** Alzheimer [tiab] OR Cognitive Dysfunction [tiab] OR Cognitive Impairment [tiab] OR Cognitive Disorder [tiab] OR Mild Cognitive Impairment [tiab] OR Cognitive Decline [tiab] OR Dementia [tiab] OR Amentia [tiab]

AND

neutrophil lymphocyte ratio [tiab] OR neutrophil to lymphocyte ratio [tiab] OR neutrophil/lymphocyte ratio [tiab] OR NLR [tiab]

**Embase**

‘dementia’/mj OR ‘Alzheimer disease’/mj OR ‘mild cognitive impairment’/mj OR ‘Alzheimer’:ab,ti OR ‘Cognitive Dysfunction’:ab,ti OR ‘Cognitive Impairment’:ab,ti OR ‘Cognitive Disorder’:ab,ti OR ‘Mild Cognitive Impairment’:ab,ti OR ‘Cognitive Decline’:ab,ti OR ‘Dementia’:ab,ti OR ‘Amentia’:ab,ti

AND

‘neutrophil lymphocyte ratio’/mj OR ‘neutrophil to lymphocyte ratio’:ab,ti OR ‘neutrophil/lymphocyte ratio’:ab,ti OR ‘NLR’:ab,ti

**Scopus**

TITLE-ABS (“dementia” OR “Alzheimer disease” OR “mild cognitive impairment” OR “Alzheimer” OR “Cognitive Dysfunction” OR “Cognitive Impairment” OR “Cognitive Disorder” OR “Mild Cognitive Impairment” OR “Cognitive Decline” OR “Dementia” OR “Amentia”)

AND

TITLE-ABS (“neutrophil lymphocyte ratio” OR “neutrophil to lymphocyte ratio” OR “neutrophil/lymphocyte ratio” OR “NLR”)

**Wos**

TS= (“dementia” OR “Alzheimer disease” OR “mild cognitive impairment” OR “Alzheimer” OR “Cognitive Dysfunction” OR “Cognitive Impairment” OR “Cognitive Disorder” OR “Mild Cognitive Impairment” OR “Cognitive Decline” OR “Dementia” OR “Amentia”)

AND

TS= (“neutrophil lymphocyte ratio” OR “neutrophil to lymphocyte ratio” OR “neutrophil/lymphocyte ratio” OR “NLR”)
